# All roads lead to the farmers market?: using network analysis to measure the orientation and central actors in a community food system through a case comparison of Yolo and Sacramento County, California

**DOI:** 10.1007/s10460-022-10345-y

**Published:** 2022-08-18

**Authors:** Jordana Fuchs-Chesney, Subhashni Raj, Tishtar Daruwalla, Catherine Brinkley

**Affiliations:** 1 Department of Human Ecology, University of California, Davis, Davis, CA 95616 USA; 2 Department of Urban and Regional Planning, University of Hawaiʻi at Mānoa, Honolulu, HI 96822 USA

**Keywords:** Alternative food networks, Centrality, Community food networks, Direct sales, Peri-urban, Short-supply chains

## Abstract

Little is known about how farms and markets are connected. Identifying critical gaps and central hubs in food systems is of importance in addressing a variety of concerns, such as navigating rapid shifts in marketing practices as seen during the COVID-19 pandemic and related food shortages. The constellation of growers and markets can also reinforce opportunities to shift growing and eating policies and practices with attention to addressing racial and income inequities in food system ownership and access. With this research, we compare network methods for measuring centrality and sociospatial orientations in food systems using two of America’s most high-producing agricultural counties. Though the counties are adjacent, we demonstrate that their community food systems have little overlap in contributing farms and markets. Our findings show that the community food system for Yolo County is tightly interwoven with Bay Area restaurants and farmers’ markets. The adjacent county, Sacramento, branded itself as America’s Farm-to-Fork capital in 2012 and possesses network hubs focused more on grocery stores and restaurants. In both counties, the most central actors differ and have been involved with the community food system for decades. Such findings have implications beyond the case studies, and we conclude with considerations for how our methods could be standardized in the national agricultural census.

## Introduction

Cross-sectoral efforts to improve human and environmental health make food systems a focus area for researchers, consumers, policymakers, and advocates. In such change-making work, partnering with central food supply chain hubs is often a strategy used to shift growing and eating practices (eg. Lubell et al. 2014). In addition to such ongoing efforts to improve food issues, recent crises in food supply highlight the need to understand supply chain connections between farms and markets. From food recalls (Roth et al. 2008), to meat packing plant closures during the covid-19 pandemic (Ijaz et al. 2021), to baby formula shortages (Abrams and Duggan 2022), to predictions of grain price spikes with the war in Ukraine (Puma and Konar 2022), farmland, markets and consumers are impacted when any point in the food supply chain is compromised. Yet, there is very little attention to identifying where such critical and vulnerable hubs in food supply chains exist. Understanding the architecture of current food networks will aid in efforts to predict and avert vulnerabilities, while assisting with long-term food system planning.

With this research, we demonstrate techniques to measure food networks, opening opportunities to ask multiple practical and fundamental research questions. In particular, this research asks two interrelated questions. First, what are the central hubs a food system? Characterizing such hubs will offer insights about power and influence on food system practices while providing practical information for food system planning. This research question speaks to a growing body of critical literature on food justice and has practical implications for organizations that have committed to Diversity, Equity and Inclusion (DEI) particularly in considering how growers of color might interface with food system hubs. Second, what are the social and spatial orientations of market relationships? Characterizing such marketing channels builds on a growing body of research in food system resilience with respect disaster management while adding practical knowledge about how to reorient food supply when major sectors are impacted (e.g., restaurant closures due to COVID-19).

This research focuses on “Community Food Systems” (CFS). The “political agenda” of a CFS is “to oppose the structures that coordinate and globalize the current food system and to create alternative systems of food production” (Allen et al. 2003). While the global industrial food system still dominates, CFS represent a collaborative and integrated network of producers, consumers, and markets that are focused on the impact of food system activities on the environment and future generations; emphasize diversity of production; and profess vocal alignment with the values of equity and social justice (Feenstra 1997; Edgar and Brown 2013). In addition, CFS typically exhibit relatively shorter supply chains than the industrial food system, connecting farmers more directly to consumers through various marketing practices, such as farmers’ markets, Community Supported Agriculture (CSA), grocery stores, and community gardens (Goodman et al. 2012), as well as forming purchasing contracts with local restaurants and institutions committed to supporting the community’s combined needs for a healthy diet, soils, and development patterns (Feenstra and Hardesty 2016). Shorter supply chains between growers and eaters in CFS help find common ground between farming practices and consumer demand, building toward a food system that meets the needs of consumers, farmers, farmworkers, and ecosystems (Hinrichs 2000). The global COVID-19 pandemic has highlighted how shorter supply chain CFSs offer greater accessibility to foods during times of crisis (Blay-Palmer et al. 2020; Raja 2020). Direct connections between consumers and farmers in CFS also provide a sense of agency that is often valued above other attributes, like organic production (Adams and Salois 2010). To raise awareness of their networks of action, CFS are often transparent, meaning markets readily promote growers, and growers, in turn, advertise the markets in which their products can be found (Trivette 2019; Brinkley et al. 2021). This transparency helps build trust in the many actors and organizations that make up the CFS (Hinrichs 2000; Brinkley 2018). The research presented in this paper is focused on such transparent food marketing relationships in CFS.

A major focus of critical studies on CFS is on the extent and efficacy of realizing diversity, equity, and inclusion ideals (Green et al. 2011; Penniman 2018; Raja 2020; Jackson 2021) and the social justice potential of CFS to reorient food systems more broadly (Alkon and McCullen 2011; Lambert-Pennington and Hicks 2016). Where 95% of US farmers identify as white (USDA NASS 2019) and very few food retail owners or managers identify as Black even in majority-Black cities (Perkins 2018), recent food justice and agricultural extension work seeks to more explicitly include growers of color. In addition, multiple studies highlight the whiteness of CFS market spaces (Alkon and McCullen 2011; Lambert-Pennington and Hicks 2016; Figueroa-Rodríguez 2019) from market organizers to shoppers at farmers markets, CFS participants tend to be white, well-educated, and affluent (Alkon and McCullen 2011; Alkon and Cadji 2020; Warsaw et al 2021), even when the market occupies space in a marginalized geography (Rice 2015). While many BIPOC-managed farms and markets may be new or new to CFS spaces, we demonstrate how a network approach can make equitable access to markets more explicit.

This research builds on national research and practice agendas for CFS. In response to growing CFS efforts and theory of change, the public sector is creating supportive funding programs and data infrastructure to chart the rise and extend of CFS. For example, the US Department of Agriculture (USDA) has added questions to the agricultural census to focus on direct marketing and local food production, finding that CFS growth has outpaced the average agricultural sector growth rate (King 2010; Low and Vogel 2011), grossing US$11.8 billion in directly-marketed local food sales alone in 2017 (Johnson 2019; Kurtz et al. 2020). The number of farmers’ markets has increased from 1755 in 1994 to 8140 in 2019 (USDA 2020) and community gardens have also proliferated with the Trust for Public Land (2021) reporting a 3000 percent increase in the number of community gardens in public parks across the 100 largest US cities: 945 in 2014 to 31,296 community gardens in 2021. More recently, the USDA has noted that sales through intermediaries such as restaurants, grocery stores, schools, hospitals, or other businesses account for two-thirds of direct sales (USDA NASS 2019), shifting a focus from exclusive emphasis on direct-to-consumer to tracing supply chains. To support such efforts, the 2018 Farm Bill increased allocations to local and regional food programming and the construction of community food infrastructure (CFI) (Johnson 2019). Simultaneously, local and regional governments are becoming more engaged in food systems planning (Brinkley 2012 and 2013; Raja et al 2018).

Despite increased data collection, funding, and planning for CFS, current methods to assess CFS are often incomplete, fail to reveal power structures and market connections, and are challenged by rapid change in practices. Acknowledging the rise of CFS, the United States Department of Agriculture (USDA) first began collecting direct sale data through the agricultural census in 2002 and produced the first Local Food Marketing Practices Survey in 2015 to “benchmark data about local food marketing practices” (USDA 2016). Though the USDA measures the number of farms selling directly to consumers in a given county, the USDA does not provide data on the ties between farms and markets within or across counties. Such information would help contextualize the CFS and its potential to pivot marketing strategies when faced with challenges, such as the COVID-19 pandemic which saw restaurants and cafeterias close for extended periods of time as consumers turned to buying food they could prepare at home. To emphasize how adjacent CFS can differ in orientation and marketing typology, this research is focused on two adjacent CFS that appear very similar according to USDA measurements of the amount of local and direct-sale food supply and marketing.

In sum, we demonstrate methodologies for understanding supply chains in CFS and global industrial food systems alike. Our methods use data the agricultural census could easily solicit. Farms know their first point of sale or donation, though not the second or third steps in the supply chain. We demonstrate how this first point of sale or donation data can be used to understand central hubs and sociospatial marketing patterns. Without such information, studies on food supply chain bottlenecks and resiliency often rely on modeled transportation routes (e.g., Lin et al. 2014 and 2019) and highlight spatial locations of network hubs (e.g., at the county or country-level) but give little practical information about the relationships between farms and outlets. Where network methods reveal power structures, they are often aspatial. For example, Trivette (2019) used a webscrape method on a single website (farmfresh.org) in New England to demonstrate how various retailing types (farms, restaurants, grocery stores, distributors) create ties/relationships across the network. He found that "retailers play a critical role in… local food systems" (2019, p. 88) based on methods that use a directed network approach to analysis. With this research, we build on such earlier efforts with methods that add spatiality to network analyses and bring nuance to the many approaches to measure centrality, identify network hubs, and assess access of different marketing typologies. In sum, to predict network functionality, growth and drivers, we argue for a mixed methods approach that is sensitive to the central actors in a network as well as the sociospatial orientation of various marketing typologies. To highlight the benefits of our methods, we contrast two adjacent county-level CFS to demonstrate their differences in CFS geographic reach and orientation, diversity of outlets, interdependency across marketing pathways, and central actors.

Moreover, our methods help frame network findings within Diffusion of Innovation Theory (DoIT) and concepts of embeddedness (Hinrichs 2000; Brinkley 2017). DoIT framework predicts how social networks help mediate the spread of information about what works or does not work for growing, packaging, and reaching new clients (Rogers 2003). Similar studies using a DoIT framing have demonstrated that the market connections between growers and purchasers lead to reinforcement of shared values and greater understanding of farming practices and policies (Lubell and Fulton 2007; Lubell et al. 2014; Hoffman et al. 2015; Aguilar-Gallegos 2015). For example, the total number of knowledge-sharing relationships that growers have correlates with their adoption of beneficial management practices (Hoffman et al. 2014). While more may be better, central hubs also act as brokers of information from disparate sides of the food system (Brinkley 2017; Pesci and Brinkley 2021). Such central hubs can be important for rapidly sharing information across the CFS (Khanal et al 2020). For example, CFS can include institutional buyers who connect with a broader base of consumers, allowing potential amplification of messaging along with marketed products. In support, Inwood et al. (2009) and Pesci and Brinkley (2021) demonstrate how chefs play a critical role in reinforcing and growing CFS; while Simin and Janković (2014) demonstrate how community food networks could expand organic agriculture practice; and Hubbard and Sandmann (2007) show how agricultural extension officers can better target educational programs and the uptake of new farming methods. Understanding the sociospatial orientation of supply chains is important for predicting where such social networks are made and how they might influence the overall success and growth of the food system. To use a simple metaphor, the general principle in network analysis is that if ‘all paths lead to Rome’, Rome is an important hub that can influence the rest of the system. Where such powerbroker central actors are located and how they direct relationships and practices may preference the values they amplify. Presumably, it mattered to the course of European development that “all roads led to Rome” and not Moscow.

As scholars focus more on social equity in the food system, a network approach can help identify such hubs as well as where historically disadvantaged growers and eaters are central or peripheral to a CFS. In continuing the “all roads lead to Rome” analogy, a network can be a “one-way street” where food flows in one direction from a farm to a market or a “two-way street” where economic exchange is embedded within shared values between farms and markets. Such considerations are important to theories of change that use embeddedness. For example, Hinrichs (2000) uses the theory of embeddedness to show how trust and political support are differently oriented for CSAs where consumers come out to the farm and for farmers markets where farmers come to the city. With network methods, we highlight the differences between a one-way, directed network and a two-way undirected network as well as its implications for embeddedness and DoIT.

In this paper, we argue that identifying hubs and socio-spatial orientations in food networks is important to understanding their overall growth and function. We use two California counties, Sacramento and Yolo, as case studies for comparing adjacent CFS. Using network analysis, we can identify the most central actors in each CFS network. We also identify market typologies and orientation. We then use this to show how hubs and market orientation impact the character and maintenance of CFS. By demonstrating multiple methods for measuring the central hubs and reach, our methods highlight the shortcomings of current supply chain data while building on earlier network approaches. We conclude by noting that the architecture of CFS market ties is important to understanding policy and practice.

## Methods

To begin, we describe the case selection and broader demographic and environmental context of the region. Then, we describe the methods used to collect data. Data gathering involved open access resources, making our methods more broadly applicable than many networks that rely on proprietary data or modeled data. Next, we describe the network statistics used to explore centrality.

### Case selection

**Fig. 1:**
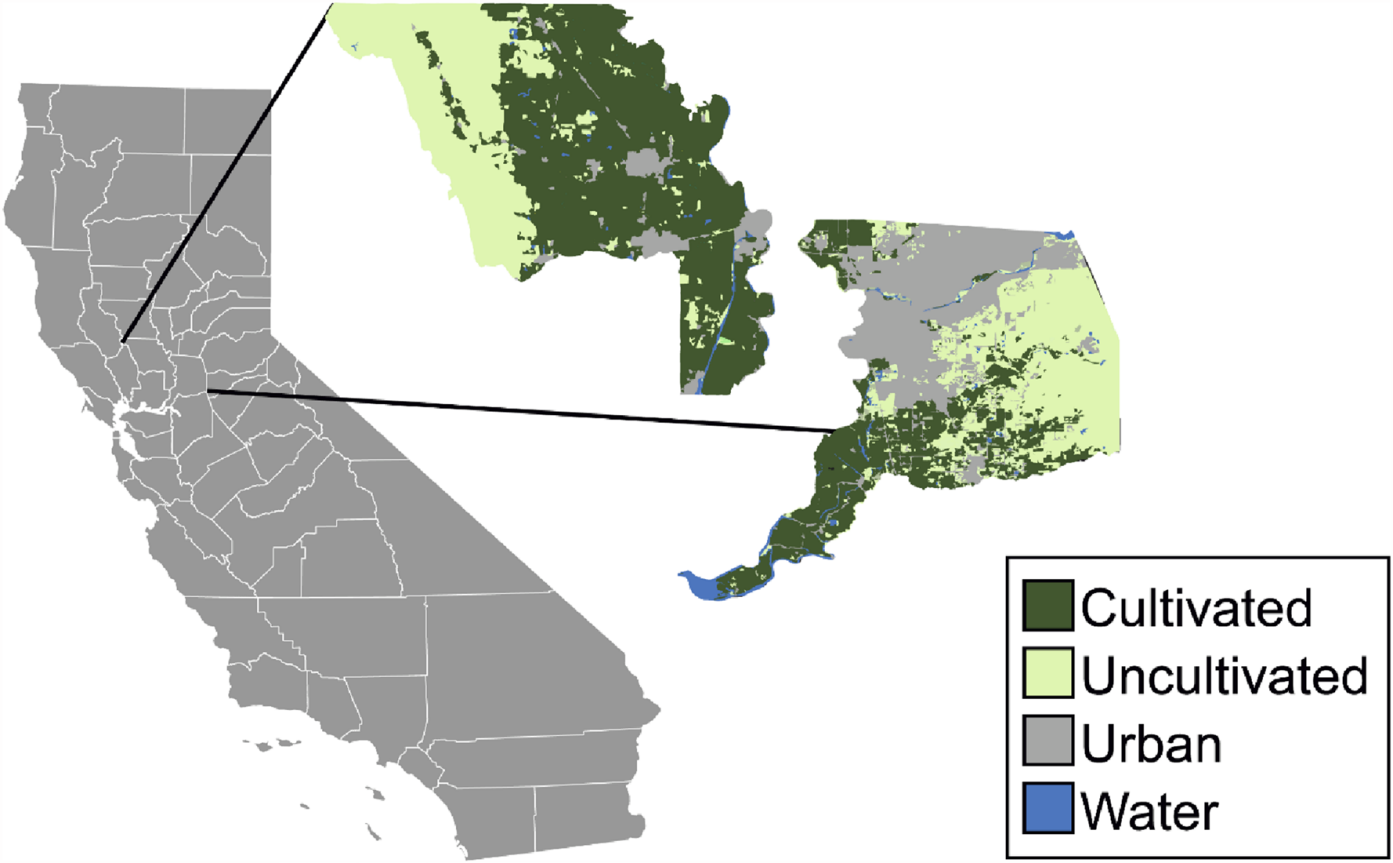
Yolo County (top left) and Sacramento County (far right) land use and position in the state of California (far left). Data source: land-use satellite imagery from the USDA National Agricultural Statistics Service (NASS)

**Fig. 2:**
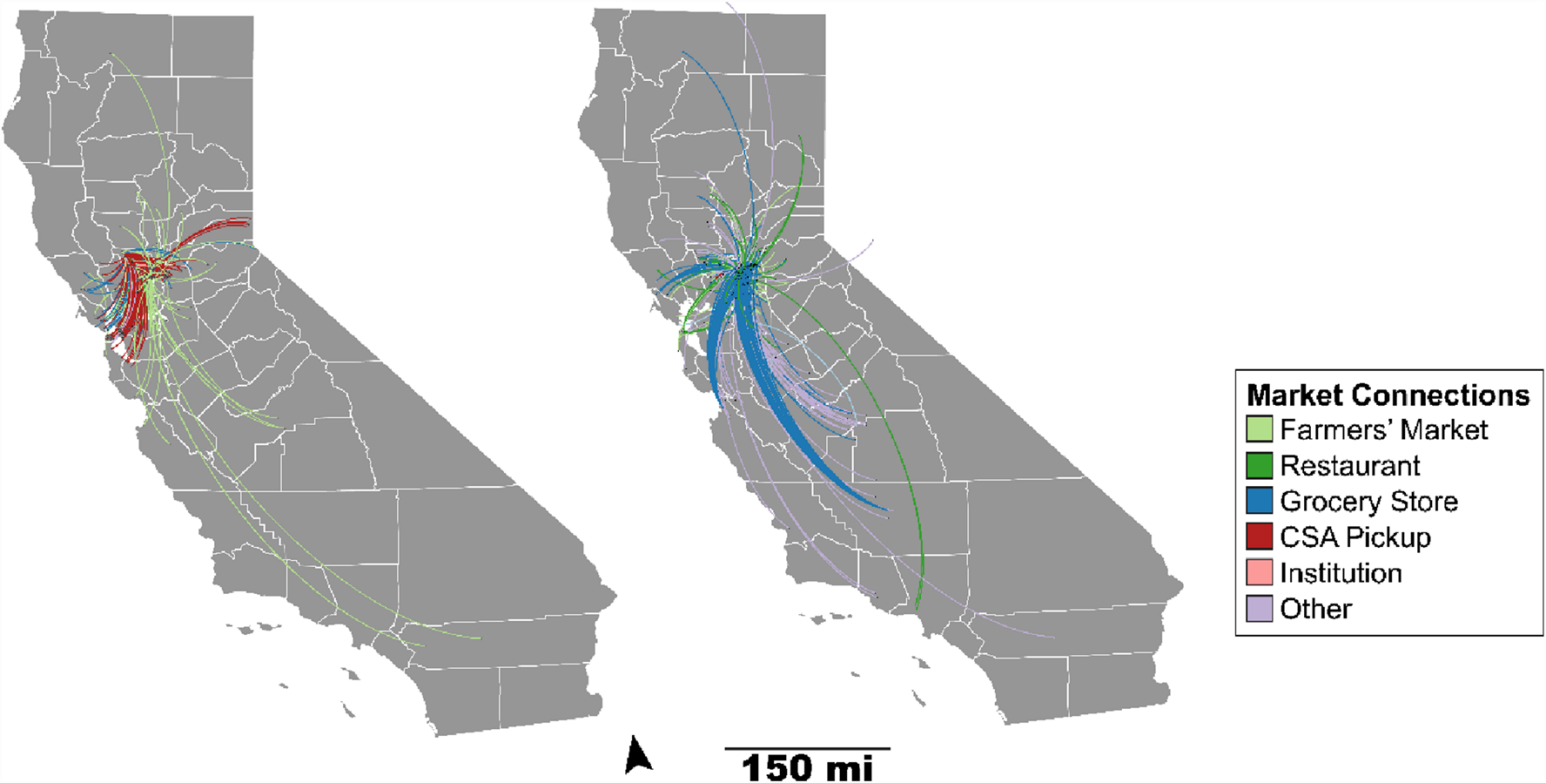
Yolo (left) and Sacramento (right) County’s community food networks represented spatially

California leads the nation in total number of farms and value of farm products sold directly to consumers, retailers, institutions, making California counties an ideal choice for this study (USDA NASS 2019). We focus one case study on Yolo County because of its longstanding, well-known ties to and dependency on agriculture. Located in Northern California, about an hour north of the Bay Area, Yolo County is home to 220,500 people, 29% of whom are non-white^1^ (US Census 2019a). About 12 percent of the population is food insecure (USDA 2020). Over 60 percent of Yolo County is farmland, with 459,662 acres actively farmed (see Figures 1 and 2; USDA NASS 2020). There are 949 farms, farmed by 1762 producers with an average farm size of 484 acres and median farm size of 50 acres (USDA NASS 2019). A majority of the producers identify as white (85.75%), and 14.25% of producers identify as non-white. Ten percent of Yolo County farms (95 farms of 949 farms) grow organically, and 14 percent (133 farms) sell directly to consumers at roadside stands, farmers’ markets, or through CSAs (USDA NASS 2019). From 2009 to 2016 the total crop revenue has risen from 462 million USD to 662 million USD (USDA NASS 2019). Notably, Yolo County is home to the Capay Valley, a patchwork of small, family-run farms growing diverse, organic produce. Yolo County’s farmland use runs the gamut from diverse organic one-acre farms to large industrial tomato processing operations. The top five crops by land cover in Yolo County are grass or land in pasture, fallowed or idle land, alfalfa, rice, and tomatoes (USDA NASS 2020). The county’s top five commodities by sales value are almonds, wine grapes, processing tomatoes, rice, and ‘organic production’ (mixed fruits and vegetables) (CDFA 2019a).

For comparison, we use Sacramento County, which sits adjacent to Yolo (Figure 1). Sacramento County is home to over 1.5 million people, with 45% of the population identifying as non-white (US Census 2019b). About 10 percent of the population is food insecure and relies on Supplemental Nutritional Assistance Program (SNAP) support from the federal government (USDA 2020). The county spans 636,000 acres, just over 40 percent of which is farmland (USDA NASS 2019). According to the 2017 USDA agricultural census, there are 1161 farms in Sacramento County, with an average farm size of 224 acres and median farm size of 13 acres. Of the 2000 producers, 14.31% identify as non-white. Two percent of Sacramento County farms (23 farms) grow organically, and 15 percent (174 farms) sell directly to consumers (USDA NASS 2019). The top five crops by land cover in Sacramento County in 2020 were grass/pasture, grapes, other hay/non-alfalfa, alfalfa, and corn, (USDA NASS 2020) and commodities by total sales value were: wine grapes, milk, nursery stock, poultry, and pears (CDFA 2019b).

### Data collection methods

We collected farm sale and donation data via a web scrape using multiple sources (Table 1) to gather information about the farm/outlet name, geographical coordinates, available contact information, sold products, county, and self-identified description of owners as BIPOC. To be included, the farm or market needed to be located in the county of interest with at least one raw product sold for human nutrition. For example, data was collected for farms in non-study counties that sell to farmers’ markets located in study counties. Using a snowball sampling technique, other farms that sold to outlets within the study county were also identified on market webpages. As a limitation, only farms with market connections advertised online are included in this research. Because this research aims to demonstrate how a similar method could be used in the agricultural census, the research did not track market-to-market sales, focusing on connections to farms.

**Table 1:** Sources consulted in compiling CFS data

Source type	Sources used
Government data	Yolo Pesticide Report 2017
Organizational data	Ecology Center FM list
Web scrape	Social networking sites
Web scrape	Google search ‘x city/county farm’, ‘x city/county farmers’ market’, ‘farm to table restaurant x city/county’, google maps search results
Web scrape	Websites of farms, farmers’ markets, grocery stores, restaurants, distributors and processors, official Yolo County website, private web portals (manta.com, agrilicious.com, localharvest.org)
Web scrape	Producer store locators on websites

To understand how representational, the data was, data on farm information was cross-checked with the USDA Agricultural census and the 2017 Yolo Pesticide Report List, which contains every farm in Yolo that was controlled by the state for pesticide activity. Of the 949 farms in Yolo County, 494 farms are on the pesticide list; 68 of those farms were found in the web scrape methods described above. Of those 68, 11 had transparent market connections. No farms were found solely through the pesticide list. In sum, no new additions were made to the data, and we concluded that our initial method captured all available online marketing data. As a result of data collection findings in Yolo County, only the web scrape method was used to capture data in Sacramento County.

Subsequent content analysis of website information and popular press news articles helped provide context about farms, farmers’ markets, market values, farming methods, and ownership. Data about BIPOC-led and supporting farms and markets were collected using a web scrape method, building on the existing farm sale and donation data. Affiliated websites and social media for each node were reviewed for mention of BIPOC self-identification. Websites were also analyzed for any mention of explicit actions or plans supporting BIPOC farmers and communities. Images were not used, nor personal experience, leading to under-counting of BIPOC engagement in the CFS. If there was no mention of self-identification as BIPOC, on a website or social media page, the node was not coded as BIPOC. After identifying BIPOC led or supporting nodes, we compared network findings with census demographic data in each county to examine the centrality of BIPOC farmers and BIPOC-supporting markets.

### Network analysis

Network Analysis was conducted using the Gephi software package. Centrality in social networks can be measured in many ways. A network can be viewed as directed, where the focus is on food moving from farms to market, or undirected, which highlights the bi-directional social relationships between countries of origin and destination. In a directed system, the emphasis is on the destinations, which receive food from multiple farms. In an undirected network, the emphasis is more on farms that participate across multiple marketing pathways.

We use three measurements to indicate centrality in a network: Degree, Betweenness Centrality and Eigenvector Centrality. It may help to think of the network as a game of Telephone, where the shortest path across the network can transmit information the fastest and most accurately. Betweenness Centrality quantifies the number of times an actor in the CFS acts as a bridge along the shortest path to connect two other actors in the network. The more a shortest path needs to go through a given node, the higher the node’s betweenness centrality, and the more influence it has on the network’s connectivity (Freeman 1977). Farms and markets that are not connected to such central lines of communication may get information/food later. We also include Eigenvector Centrality, which measures the relative scores to all actors in the network based on the concept that connections to more centrally located actors contribute more to the score of closely related actors. In this sense, Eigenvector Centrality considers ‘who your friends are’ as important to your own centrality. Being associated with groups closer to the center of the network likely helps you receive information faster and more accurately while providing feedback and shaping the overall network more directly. Last, Degree measures the total number of connections a node has, not necessarily its positionality in the network. A farm could sell to hundreds of outlets that are not used by any other farm in the CFS, representing opportunities to partner and a broad array of novel actors. Yet, having so many partners does not necessarily make that farm central to the network if it is disconnected from the rest of the farms and markets.

To add qualitative findings, we used a document review of websites and news articles related to central actors in the network.

## Results

First, we describe how representative our results are based on USDA agricultural census data. Next, we describe how a network approach reveals new information about county marketing typologies and reach that is not found in the agricultural census. Then we use the network analysis to identify the most central actors in each CFS, detailing how each centrality measure differs to help researchers new to network analysis better understand the implications of various centrality measurements used. Last, we compare the positionality and access of BIPOC-led farms and markets with those of central actors to understand equitable access in each CFS.

Food systems are not neatly bound by county boundaries. First, we show how some data points align with USDA Agricultural census figures while others differ due to the data gathering methods. For example, most farmers’ markets advertise online while many farms do not. Thus, USDA assessments and our methods for assessing the number of farmers’ markets in CFS align but differ on the total farms reported. The USDA reports six farmers’ markets in Yolo County and 27 in Sacramento. We also find six farmers’ markets in Yolo County but note that the Yolo County CFS connects with a total of 26 farmers’ markets. In part, this discrepancy between USDA counts and our methods occurs because many Yolo county farms participate in farmers’ markets outside the county. In the case of Sacramento, the primary farmers’ market operator does not list information about their market connections. Because our method emphasizes transparent connections, we did not include the markets that did not list connections with their farms.

Our method provides an undercount of a CFS. Figure 2 (left) shows 68 farms (39 of which are located in Yolo County) and 446 market connections representing the CFS in Yolo County. The USDA data reported 136 farms in Yolo County that market local food and 27 that market through CSA, indicating that findings represent 29% of the farms that reported direct marketing. Similarly, in Sacramento, the USDA reports 174 direct-sale farms with 19 that sell directly through CSAs; our data shows 222 farms in the Sacramento network with 61 of those farms located within the county. The Sacramento CFS represents 35% of the farms that direct market according to the USDA Agricultural Census. These differences between the USDA dataset and the web scrape data are likely attributable to the lack of online presence for many of the farms participating in direct sales, especially those that sell through roadside stands. Additionally, the agricultural census measures direct sales of processed foods and wine, whereas our method tracks only raw, unprocessed farm products.

### Spatiality

Though USDA data is silent on how CFS connect across communities, our methods reveal important distinctions between the CFS of two adjacent counties. Yolo County’s CFS is tightly connected with surrounding counties and the Bay Area as shown in Figure 2. Our finding confirms earlier economic studies (Hardesty and Christensen 2016) that noted the connections between Yolo County farms and Bay Area markets and consumers. While Sacramento County is adjacent to Yolo County, it is far more connected to California’s Central Valley producers who sell through grocery stores in Sacramento County (Figure 2). This finding is presumably because Sacramento has a larger population and can draw more food into the county for sale. In addition, only 17 nodes overlap across both counties, further indicating that the county networks differ. Of these 17, 12 are farms that sell in both counties; two are school districts that draw from farms in both counties; one is a farmers’ market; another a restaurant and another a grocery store. While both counties have a similar number of farms that sell directly to consumers or support CSA sales (Table 2), Yolo County has more agricultural land and fewer producers. The spatiality of each county’s CFS likely shifts its priorities and focus areas. For example, as Yolo has deeper ties to San Francisco, one of the nation’s largest and wealthiest cities with nearly no farming, connections with Bay Area eaters might focus more on diet and consumer concerns. Conversely, Sacramento’s connections to the Central Valley may generate more of a shared understanding for farming communities that sell food near the state’s capital.

**Table 2:** Participants in the Yolo and Sacramento County CFS

Contributors	USDA Yolo	Web Scrape Yolo	USDA Sacramento	Web Scrape Sacramento
Farmers’ Market	6	6 (26 total)	27	14 (19 total)
Grocery Store	44	10 (55 total)	275	99 (105 total)
Restaurant	144	5 (40 total)	981	32 (46 total)
Farm	136 (27 CSA)	39 (68 total)	174 (19 CSA)	61 (222 total)
Institution	1	6 (8 total)	1	10 (11 total)
Other	NA	34 (189 total)	NA	18 (21 total)

The web scrape data displays total network participants in parenthesis, with in-county contributors preceding. The majority of the sites in the ‘other’ category in Yolo County are CSA pick-up locations and distributors in Sacramento County. All USDA-related data was obtained from the Food Atlas. Data for the different contributor categories are from different years owing to differences in data collection and availability by the USDA. USDA farmers’ market information is from 2018. USDA farms with direct sales data is from 2017 (with farms that have CSAs in parentheses); USDA grocery store and full-service restaurant information is from 2016. The only institution noted in USDA data is Farm to school programs, with the latest figures reported in 2015

**Fig. 3:**
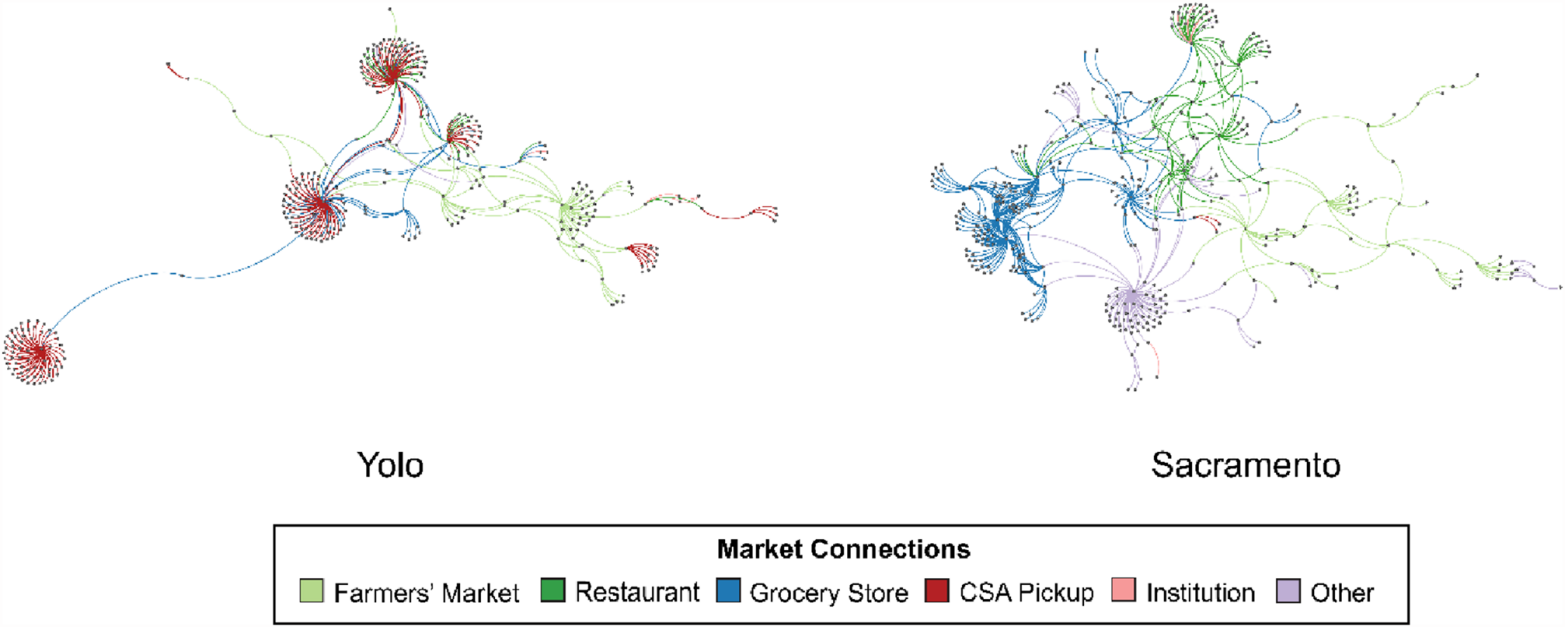
CFS network of Yolo (left) and Sacramento (right) counties. Network layout: Yifan Hu

The networks also differ in sales typologies (Tables 2 and 3). Three-quarters of Yolo County’s CFS consists of ties through CSA sales and farmers’ markets (Table 3), indicating Yolo County’s CFS capacity to redirect food sales from restaurants and institutions as they scale back during the COVID-19 outbreak. Importantly, there are many farmers’ markets in the Bay Area, demonstrating the close relationships between Bay Area consumers in supporting Yolo County farmers. Grocery stores are the third most prominent outlet connection for Yolo County farms (Table 3). Conversely, most of Sacramento County’s connections are through grocery stores, followed by farm sales to restaurants (Table 3, Figure 3). While a few farms specialize in one marketing typology (e.g., CSA sales), most farms have a mixture of marketing strategies (Figure 3), further generating flexibility if there is a need to scale up or down one marketing pathway. This data does not include the total weight, value or number of products sold, nor consumers served. Thus, the ties represent farm connections to markets, but not necessarily the strength nor dependency on the market tie.

**Table 3:** Marketing connections (edges) for Yolo and Sacramento Community Food Systems

Connection types (edges)	Yolo	Sacramento
Farmers’ Market	110	73
Grocery Store	65	262
Restaurant	31	100
CSA Pickup	210	8
Institution	9	14
Other	21	152

The Yolo network “other category” includes farm stands, u-picks, distributors, and online sale. The Sacramento “other” category includes farm stands, box schemes, catering, farm-to-farm sales, non-grocery store, online sales, distributors, and u-picks. The u-pick and farm stand relationships are self-loops that have been removed in the network visualizations (Fig. 3)

### Most connected farms/institutions

Because an actors’ position in a social network denotes the ability to receive or share knowledge (or in the case of CFS: food), we visualize the network and use centrality statistics to understand which farms and markets are central gateways to more disparate parts of the network. Centrality is a measurement of how connected a node is to the rest of the network. Nodes with high centrality have the potential to reach across the network, broker new information, and coordinate the spread of new ideas.

### Network characteristics

Our network characteristics are comparable to other CFS networks reported in the literature. For example, Trivette (2019) explored a multi-state CFS network with 1323 nodes with a directed network density of 0.001851. A density of 0 indicates a network of completely disconnected nodes while a density of 1 indicates a fully connected network. The county networks we present are 3–6 times more connected when compared to Trivette’s (2019) dataset. Similarly, a smaller network diameter indicates that the network can be traversed in a few connections (Brinkley 2018) and is sometimes called a ‘small world network’ (Travers and Milgram 1967). A larger network diameter is characteristic of a lattice structure and requires more connections to traverse. Like Trivette’s (2019) CFS, our county networks are low-density, small-world networks (See Table 4).

**Table 4:** Network characteristics

Connection types (edges)	Yolo	Sacramento
Total nodes	386	424
Total Edges	446	609
Average Path Length (undirected)	4.5	4.6
Network Diameter (undirected)	11	12
Network Density (undirected)	0.006002	0.006791
Network Density (directed)	0.003001	0.003396

Some contributors are more central to the network based on their market connections. These central hubs bring together products from multiple farms or sell through a variety of markets. We start by exploring the degree (total number of connections), a network feature that will not change based on network edge directionality. For each actor/node, we offer background found in content review (Appendix 1).

Despite counties being adjacent, there was little overlap in the topmost central actors regardless of which network centrality measure was used. For Yolo County, the actors with the highest degree are Full Belly Farm, Riverdog Farms, Terra Firma Farm, Davis Farmers' Market, and Say Hay Farm (see appendix 1 for description). For Sacramento County, the actors with the highest degree are General Produce, Bolthouse Farms, Ocean Mist Farms, Niman Ranch, and Aldon’s Leafy Greens.

Centrality, as measured by degree, in Yolo County’s food network is dominated by family farms, apart from the Davis Farmers’ Market, which espouses similar agro-ecological values as the farms. Yolo County farms with the highest degree all sell through CSAs and maintain organic certification and sustainable agricultural practices, demonstrating the values of the CFS. High degree Yolo County farms also sell across the Bay Area through farmers’ markets, and in Yolo and Sacramento counties through local retail stores, restaurants, and prepared food vendors. In contrast, with the exception of Aldon’s Leafy Greens, degree centrality in the Sacramento County food network is characterized by farms that sell more nationally than locally or regionally. Most of what is grown by central hubs in the Sacramento CFS is marketed outside the immediate area, and there are few avenues to directly obtain products from these farms within the county. The contrast between central hub market orientation and the rest of the Sacramento CFS with its focus on central valley farms selling into the Sacramento area may underscore a disconnect between the national food suppliers and the farm-to-fork ethos espoused in branding materials for the capitol city. In summary, degree is a measure of “popularity” that emphasizes total connections over how those connections relate to the network. A node’s degree offers information on CFS functionality that the USDA’s agricultural census does not capture.

Next, we explore network centrality as measured by betweenness and eigenvector for undirected networks. Rockridge Market Hall is the only venue in the top betweenness centrality rankings for Yolo County that is located outside of the county, again reinforcing the ties between Yolo growers and Bay Area eaters. Similarly, eigenvector centrality on the undirected Yolo network introduces only one actor not located in Yolo County: Sacramento Natural Foods, located in Sacramento County. Interestingly, this is the only actor to occupy a top-five ranking across both networks, perhaps indicating an important convening spot for joint Yolo-Sacramento food systems efforts. For Sacramento, the top venues with the highest betweenness centrality as measured with an undirected network include one restaurant, the Kitchen, which gained a Michelin star shortly after the “Farm-to-Fork Capital” slogan for Sacramento was adopted (The Kitchen 2021). When using eigenvector to measure centrality on an undirected network, Sacramento’s network includes a grocer and otherwise top entries overlap with highest degree farms (Table 5). In summary, like Trivette (2019), we find that high degree overlaps with other undirected centrality measures.

**Table 5:** Network centrality for the top five actors

Rank	Yolo- total degree	Yolo- betweenness	Yolo- eigenvector undirected	Yolo- eigenvector directed	Sacramento- total degree	Sacramento- betweenness	Sacramento- eigenvector undirected	Sacramento-eigenvector directed
1	Full Belly Farm	Full Belly Farm	Full Belly Farm	Davis Farmers’ Market	General Produce Company	General Produce Company	Bolthouse Farms	General Produce Company
2	Riverdog Farm	Riverdog Farms	Riverdog Farm	San Rafael Farmers’ Market	Bolthouse Farms	Bolthouse Farms	Ocean Mist Farms	The Waterboy
3	Terra Firma Farm	Davis Farmers' Market	Terra Firma Farm	Veritable Vegetable; Downtown Palo Alto Farmers’ Market; Downtown Berkeley Farmers’ Market; South Berkeley Farmers’ Market; Sacramento Natural Foods Coop	Ocean Mist Farms	The Kitchen	General Produce Company	Onespeed Pizza
4	Davis Farmers’ Market	Terra Firma Farm	Say Hay Farm		Niman Ranch	Niman Ranch	Niman Ranch	Sacramento Natural Foods Coop
5	Say Hay Farm	Rockridge Market Hall	Sacramento Natural Foods Coop		Aldon’s Leafy Greens	Aldon’s Leafy Greens	Safeway—Crocker Drive	Seka Hills

Six organizations ranked as the third most central by eigenvector centrality in Yolo’s directed network

When using a directed network for Yolo County, the most central venues by eigenvector centrality are largely farmers’ markets in urban areas outside the county. Sacramento’s directed network gives more preference to restaurants, but also includes a grocer (Sacramento Natural Foods Cooperative) and farm, Seka Hills. This farm and market is owned and operated by the Yocha Dehe Wintun Nation and farms with sustainable practices (Seka Hills 2016) providing smaller grocery store outlets with fresh olive oil, vegetables and nuts (Seka Hills 2016). In summary, restaurants are central to Sacramento and farmers’ markets to Yolo County’s CFS (Tables 3 and 5, Fig. 3) when measuring centrality based on directed networks.

In summary, the undirected centrality network approach generally emphasizes farms as central over markets. Indeed, many of the most central farms have played a large role in their local food systems and agricultural policy for many decades. For example, Full Belly Farm was established in 1985 and is used as a USDA (2020) case study on climate ready agriculture. Similarly, Niman Ranch was established in 1970 and has been an early partner in Alice Water’s Chez Panisse farm-to-table efforts (Pesci and Brinkley 2021). This methodological distinction between directed and undirected networks will be important for future research to note in reporting findings about food systems as centrality has implications for power relations that are capable of directed embedded values and the diffusion of new ideas.

Most striking is how little overlap there is between the CFS in both counties (Table 5). While some farms and markets serve both counties, Yolo County is oriented spatially and socially toward the Bay Area while Sacramento County is tied spatially and socially with the Central Valley. Both networks have organizations at their centers, no matter which centrality measure used, that have been in operation for several decades and are actively influencing growing and consuming practices.

In sum, network methodologies allow researchers and practitioners to reveal how differently organized and oriented food systems may be based on the make-up of their central actors. In comparing undirected and directed networks along with a variety of centrality measures, we show how different methods will lead to alternate conclusions about centrality. Such nuances can be avoided in future research by reporting on all centrality scores or providing clear theoretical rationales for why particular centrality measurements and network directionality are used. In particular, we highlight that where directed networks are used, they will yield findings that preference markets as central to food systems above farms. In Yolo county, four of the five most central actors were farms in the undirected network compared to the five central markets in the directed network. Similarly, Sacramento shows four of the five most central actors as farms in the undirected network, but four of the five most central are farms in the directed network. As the USDA highlights the role of intermediaries in growing food systems and network research highlights “the important role food retailers play in the overall vibrancy of local food exchanges” (Trivette 2019), our findings remind scholars that importance and centrality is a matter of the methods used to measure such centrality, thereby refocusing the theory on farms as well as markets.

### Centrality and equity

**Fig. 4:**
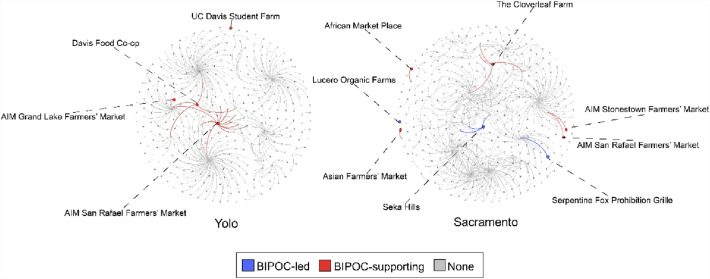
BIPOC-led and BIPOC-supporting organizations and their ties in both Yolo and Sacramento County CFS. Network Layout: Fruchterman Reingold. More central nodes are more central in the network visualization

Next, we use website and content analysis on central actors in the CFS to demonstrate how aspects of equity can be considered in a network approach. Only three growers and market managers self-identified as non-white on their public-facing website though the USDA reports 429 self-identified BIPOC growers the bi-county region (USDA NASS 2019) and the counties are home to a diverse population base: 45% and 29% of people in Sacramento and Yolo Counties respectively identify as non-white (US Census 2021). We find that two of the three self-identified BIPOC-owned farms and markets are peripheral to their CFS; i.e., they do not occupy central hubs (Fig. 4). However, BIPOC-led farms and markets on the periphery of the network are creating supporting networks of their own. For example, the African Market Place, founded in 2015, is a market for Black farmers and producers. The Market Place is a part of a broader effort to uplift Black culture and community in Sacramento. Similarly, the Asian Farmers’ Market in downtown Sacramento provides both a market for Asian farmers as well as consumers looking for Asian produce not commonly found elsewhere.

Of the three self-identified BIPOC-led organizations in the bi-county region, one is central. Operated by the Yocha Dehe Wintun Nation, the Seka Hills farm and tasting room is the only self-identified BIPOC-owned and managed organization across all centrality rankings. Though located in Yolo County, Seka Hills is not ranked as centrally in the Yolo County CFS, but is in Sacramento’s CFS. Our network methods help highlight the positionality of this BIPOC-led organization within its home county and adjacent CFS, bringing nuance to its role in setting food system policy and practice.

In addition, many CFS actors are focused on addressing equity though they may not identify growers or market managers of color. For example, the Agricultural Institute of Marin (AIM), operating three markets in the Yolo network and two markets in the Sacramento network, has created a “Path to Racial Equity” plan in which they acknowledge the role of racism in food systems and lay out specific steps for all tiers of their organization to make their organization and markets more equitable. Interestingly, there are AIM markets in both Yolo and Sacramento County networks, but in Yolo County, the markets are central to the network, and in Sacramento County, the markets are peripheral. This speaks to how different the neighboring counties’ networks are and supports our findings that Yolo County’s network is heavily oriented toward the Bay Area.

## Limitations

Prior research (Hoffman et al. 2015) has noted the importance of recognizing social networks for extending agricultural extension knowledge through trusted channels. This research adds the perspective of marketing channels while drawing attention to a method of data gathering and analysis. The methods offer a characterization of a CFS, noting spatiality, composition, and centrality of farms and markets. Many farmers may not yet have any online presence and are not represented in our data. The weight of market ties in both pounds of food and social importance for policy setting can offer further insights into how a county can mobilize across the agricultural sector on a variety of topics. Additionally, our research captures only a static, point-in-time measure of these networks; further research is needed to accurately capture how CFS networks evolve and change over time. Indeed, the ever-evolving nature of CFS means that in subsequent analysis, many of the nodes in the original dataset had no website or a website that was no longer functioning. Last, many of the farms and markets that are BIPOC-led or supporting do not self-identify as such in online profiles, leading to undercounting in our dataset and analysis. Nevertheless, network visualizations help make access to CFS markets and knowledge networks explicit, and highlight opportunities for increasing equity, while more qualitative work in surveys, interviews and content analysis is needed to understand power relationships.

## Discussion

The methods we present in this comparative case study highlight how the central actors and socio-spatial orientation of each CFS may differ even where the numbers of engaged actors are similar *and* the counties adjacent. In particular, the network in each county is not bounded by jurisdictions but spans multiple counties, including both urban and agricultural landscapes. Our findings align with the USDA’s report on the importance of intermediaries as conduits of direct sales (Low and Vogel 2011); our data is representative of general trends in the number of engaged actors in a county food system (Table 2); and our network is similar in architecture to CFS networks in other parts of the country (Trivette 2019). Yet, though both counties are spatially proximate and appear similar in the USDA Agricultural Census, their CFS are oriented toward different regions, and are thus involved in differing marketing relationships and food system policy conversations. Based on our findings, we would expect that Yolo and Sacramento Counties will experience differing growth and resiliency impacts to their CFS due to having different central actors that are interacting through different marketing channels, with different clientele, in differing regions. Our findings would predict that Yolo County’s CFS will continue to grow in step with the Bay Area, while Sacramento’s CFS may have a bifurcated growth pathway that is tied both to national-reaching agrofood businesses and to Central Valley farms. As a result, our findings demonstrate how applying such network methodologies to food systems will be important to understanding the unique evolutionary paths of interrelated food systems.

Further, this study also adds caution for future network studies by demonstrating how the use of directed or undirected networks may prejudice findings. For example, the directed network approach emphasizes the role of markets while the undirected network approach emphasizes the role of farms as central to the network. While intermediary markets are important in food networks (eg. Trivette 2019), farms may be just as important to the innovation of new farming and marketing practices like CFS. A directed network approach may overplay how central markets are to CFS. Indeed, many of the most central farms at the heart of both networks have been involved in their food systems for over half a century and played high-visibility roles in setting agricultural policy. Understanding market and farm influence is just as important to the theories involved in CFS as to the practice of intervening in food systems.

Diffusion of Innovation Theory (DoIT) draws attention to the rise spread of novel practices as mediated through social networks (Rogers 2003) and embeddedness (Hinrichs 2000) highlights that values and economic systems are often intertwined. Our study fits such theories with empirical methods to identify powerbrokers and overall network sociospatial orientation. To this end, both counties have *different* long-established nonprofits, farms, and markets at the heart of their CFS regardless of the network directionality or centrality statistic used. This finding would indicate that different values may be embedded across the Yolo and Sacramento networks and that novel approaches may diffuse differently due to the different cast of powerbrokers at the heart of each network. As such, the methodological framework we built with this research has implications beyond the northern California CFS case studies in allowing deeper inquiries about power and influence in food systems. If measured over time, a sociospatial network approach will allow future research to ask whether network orientation and growth occurs at the direction of central hub organizations or from more peripheral organizations, ultimately asking whether value and economic embeddedness is more or less functionally important for particular consumers and allied institutions.

Such network approaches to food systems open possibilities in scholarship and practice that center equity. Namely, both high degree and centrality (undirected and directed) is a sign of seniority and importance in the CFS, having operated in the space for a long time and successfully cooperating with and across multiple other network actors and institutions. To this end, many of the markets highlighted in this research have had historic emphasis on low-income and smaller-scale growers, helping them stay in farming by gaining a market for their food in high-end restaurants and downtown markets. Often, such marketing focuses in CFS emphasize sustainable and agroecological growing methods over considerations like social and racial equity. For example, Alkon and McCullen’s (2011) study of farmers’ markets found that the Davis Farmers’ Market, a central hub in the Yolo County CFS, consisted predominantly of white farmer-farm operators and their white non-farmworker employees, rendering people of color who predominantly grow and pick food on these farms invisible (Alkon and McCullen 2011). Ten years since that study, and a review of the Davis Farmers’ Market website for key terms such as justice and equity yields no results. Instead, the market’s mission statement frames only environmental, economic, and nutritional benefits: “to educate and engage the public about nutrition, sustainable agriculture, and the economic value to our area of buying locally grown food and locally sourced products directly from growers and artisans” (Davis Farmers’ Market 2021). With a shift toward food justice, a network approach allows communities to see which organizations are central to their CFS and where to advocate for greater inclusion (and centrality) of historically disadvantaged growers and marketers in both mission and action.

Networks offer the ability to visualize such values and identify practical, local solutions. For example, farms with robust online platforms, like Capay Organic’s online ‘Farm Fresh to You’ are incorporating products from other farms into their home delivery boxes, opening opportunities for partnership with other sustainable growers and farmers of color. The network approach in this research identifies where such connections could be made and strengthened should policymakers and entrepreneurial efforts wish to place more emphasis on equity. Similarly, identifying and celebrating BIPOC growers, such as the Yocha Dehe Wintun Nation, who are central to food networks can enable consumers to up-vote racial equity and indigenous food system ownership with their purchases. Importantly, our methods underestimate the involvement of growers of color in the food system based on the web scrape technique. Several farmers of color did not list their identities as such on their farm websites. Going forward, scholars and practitioners working on anti-racist strategies for CFS can use similar network methods to identify central hubs and sub-networks to intentionally engage in food justice. Future work could also pair such network approaches with interviews and focus groups to ground truth findings and better understand why and how farmers of color may have better success in gaining market access outside their home county or by seemingly building their own parallel networks.

Last, methods like those we present can help answer such questions at the national scale, particularly if the USDA Agricultural Census begins to collect first point of sale or donation data. Such information would enable food supply network analyses that could trace contamination, identify hubs that lead to vulnerability in the overall food system, and quantify market access. We hope that this study has helped demonstrate the potential of a national dataset, particularly because data assembly of public information at the county level is a costly endeavor that will limit reproducibility.

## Conclusion

With this research, we highlight a method of identifying a CFS reach, network architecture and central actors by using publicly available data and social network analysis. Where the USDA agricultural census would not detect many differences across the Yolo and Sacramento County CFS, a network approach highlights differences that are of importance to larger theoretical questions about food systems as well as practical concerns for food supply. We demonstrate that even adjacent counties can have different CFS network structures, with little overlap in central actors, and be spatially oriented towards different regions and markets. In network approaches, if “all roads lead to Rome”, then Rome is an important coordinating hub in the overall network. With this research, we show how the food ways in two adjacent counties lead to different hubs. We also show how the market connections in a food system are likely ‘two-way streets’ based on the centrality of both farms and markets in food networks, depending on the network approach taken to measure centrality (directed or undirected). In addition, we find that regardless of the network directionality used, farms and markets that are central to their CFS have been in operation for decades and are often involved in food and agricultural policy with a focus on growing support for agroecological practices.

Such findings have implications for coordinating outreach across the food system. For example, we demonstrate a method for assessing equitable market access for BIPOC growers, revealing how an indigenous-run farm is a central actor in one CFS though it is located in the neighboring county. Such differences in central actors and sociospatial orientations likely constrain and direct CFS growth, impacting overall CFS equity, access and resiliency. Our methods can be used by researchers and policymakers to assess practical efforts towards justice, equity and resilience in local and global food systems.

Last, we suggest the USDA collect first point of sale or donation of food data from farms in the agricultural census. Such an effort could correct for current shortcomings in USDA data where local and national food system data is not connected to supply chain considerations, thus limiting the ability to predict where bottlenecks could occur nor model future growth and change. With two case studies, this research demonstrates the value of a national dataset. Further, if the USDA Agricultural Census were to employ the first point of sale or donation question combined with farm manager demographic data, a more representative description of equitable access to markets could be achieved along with information about the central hubs and market orientations.

## Yolo County

- **Full Belly Farm** is a 450-acre organic farm in the Capay Valley that has been in operation since 1985 (Full Belly Farm 2021). The farm grows 80 different crops and keeps heritage animals, incorporating them into the farm’s ecosystem (Full Belly Farm 2021). Their produce can be found throughout the Bay Area and Sacramento region, at farmers’ markets, grocery stores, restaurants and a CSA that serves the Bay Area and Sacramento region (Full Belly Farm 2021). They are award-winning for their sustainability efforts, including solar power, crop diversification and rotation, native habitat restoration, and support of beneficial insects and pollinators (USDA California Climate Hub 2020).
- **Riverdog Farm** started as a two-acre organic vegetable farm in Napa County in 1991 and moved to the Capay Valley in 1996 (Riverdog Farm 2021). It is now 450 acres. They grow a variety of crops as well as pasture-raised chicken (Riverdog Farm 2021). They have sold at the Berkeley Farmers’ Market since their inception and run a CSA that serves the Bay Area and Sacramento region as well as selling through wholesale and retail outlets (Riverdog Farm 2021). Together with Full Belly Farm, Riverdog Farm has partnered with University of California Cooperative Extension officers to obtain grants (Davis Enterprise 2016) and have seen CSA memberships increase since the coronavirus pandemic (Daily Democrat 2020).
- **Terra Firma Farm** is on 200 acres near Winters, in the southwest of Yolo County (Terra Firma Farm 2021). They operate a CSA and deliver throughout the Bay Area as well as provide produce to local co-operative grocery stores and nearby restaurants in Sacramento and the Bay Area (Terra Firma Farm 2021). Terra Firma has been farming in Winters, CA for 25 years and still employ some of their original staff (Terra Firma Farm 2021).
- **Davis Farmers’ Market**, located in the City of Davis, was established in 1976 and was one of the first markets in the renaissance of farmers’ markets in the US, as well as one of the first producer-only markets, a requirement that is now standard at farmers’ markets across the state (Gumprecht 2008). Today the Davis Farmers’ Market has expanded to support four markets in the area at UC Davis and at Sutter Medical Center (Davis Farmers Market 2021)
- **Say Hay Farm** is a 50-acre farm in Yolo County, CA that raises certified-organic vegetables, melons, oranges, and eggs in an ecological manner (Center for Agriculture and Food Systems n.d.). Say Hay sells through a variety of venues, including Grand Lake Farmers' Market, The Local Butcher Shop in Berkeley, and through distributors like Good Eggs (Daruwalla et al. 2021).
- **Rockridge Market Hall**, located in Oakland, was founded in 1986 (Rockridge Market Hall 2021)
- The **San Rafael Farmers’ Market** is operated by the Agricultural Institute of Marin, a Bay Area nonprofit founded in 1986 with the mission of elevating local food and farmers through their eight established farmers’ markets (Agricultural Institute of Marin 2021).
- **Veritable Vegetable** is a women-owned and led organic produce distribution company based in San Francisco, California and established in 1974 (Veritable Vegetable 2020).
- **The Downtown Berkeley Farmers’ Market** has been managed by the Ecology Center since 1987 (Ecology Center 2021). The Ecology Center is a Berkeley-based nonprofit that was founded in 1969 with the mission of improving the health and the environmental impacts of urban residents through demonstration projects, like the four farmers markets it manages (Ecology Center 2021).
- **The downtown Palo Alto Farmers’Market** is a nonprofit that was originally founded in 1981 (Downtown Palo Alto Farmers’ Market 2021).
- **The Sacramento Natural Foods Cooperative** was opened in 1972 as a food buying club, and transitioned a year later into a cooperative (Sacramento Natural Foods Cooperative 2021).

## Sacramento County

- **General Produce Company** is a third generation owned and operated distributor focused on sustainable business practices located within Sacramento County (General Produce 2021). Founded in 1933 by a Chinese immigrant to California, sourcing and selling fresh fruits and fish in Sacramento, they now source produce from local farms and export fruits and vegetables throughout California, Nevada, and Southern Oregon (General Produce 2021).
- **Bolthouse Farms** is a large-scale farm headquartered in Bakersfield, CA that sources from many farms who contract with the company (Bolthouse Farms n.d.). Bolthouse Farms provides Sacramento County’s grocery stores with various mixed greens and vegetables (Daruwalla et al. 2021).
- O**cean Mist Farms** is a specialty grower of artichokes that ships their vegetables nationwide from their headquarters in Castroville, CA and sells to several Bel Air and Walmart Neighborhood Market stores (Ocean Mist 2021).
- **Niman Ranch** headquartered in Colorado and supports a network of small family ranchers (Niman Ranch 2021). While headquartered in Colorado, Niman Ranch started as as a family farm in Northern California (Daruwalla et al. 2021).
- **Aldon’s Leafy Greens** is a Controlled Environment Agriculture (CEA) farm that specializes in microgreens and grows other various dense lettuces (Aldons Leafy Greens 2021). Aldon’s sells through the Tahoe Food hub and over 30 local restaurants (Aldon’s Leafy Greens 2021; Daruwalla et al. 2021).
- **The Sacramento Natural Foods Cooperative** was opened in 1972 as a food buying club, and transitioned a year later into a cooperative (Sacramento Natural Foods Cooperative 2021).
- **The Kitchen** is a Sacramento City-based restaurant that gained a Michelin star shortly after the “Farm-to-Fork Capital” slogan for Sacramento was adopted (The Kitchen 2021). The restaurant uses their website to write blog posts on where they source ingredients from, and often showcases producers they work with throughout the Sacramento Valley as well as all of California (The Kitchen 2021).
- **Safeway**- Crocker Drive. Safeway is a national chain grocery retailer (Safeway 2021).
- **The Waterboy** is a midtown Sacramento restaurant in operation since 1996 (The Waterboy 2020) The head chef and owner of Waterboy, Rick Mahan, opened **OneSpeed** Pizza in 2009, using a similar model of highlighting ingredients from regional farms (OneSpeed Pizza 2020).
- **Seka Hills**, in neighbouring Yolo County, provides the smaller grocery store shoppers with fresh olive oil, vegetables and nuts (Seka Hills 2016). They are owned and operated by the Yocha Dehe Wintun Nation and farm with sustainable practices (Seka Hills 2016).

## Abbreviations

AIM: Agricultural institute of marin
BIPOC: Black, indigenous, people of color
CFI: Community food infrastructure
CFS: Community food systems
CSA: Community supported agriculture
DEI: Diversity, equity and inclusion
DoIT: Diffusion of innovation theory
SNAP: Supplemental nutritional assistance program
USDA: United States department of agriculture

## Footnotes

1 We use ‘non-white’ because this is how the census defines this self-reported demographic category. Defining BIPOC or people of color as ‘non-white’ is problematic because of complex, intermingled histories and structural racism. Nonetheless, the term and population statistics associated provide descriptions of the county demographics.
